# Applying the workload indicators of staffing need (WISN) method in Namibia: challenges and implications for human resources for health policy

**DOI:** 10.1186/1478-4491-11-64

**Published:** 2013-12-10

**Authors:** Pamela A McQuide, Riitta-Liisa Kolehmainen-Aitken, Norbert Forster

**Affiliations:** 1 IntraHealth International, Eros, PO Box 9942, Windhoek, Namibia; 2 Calle Obispo Hurtado 24–9 F, Granada 18004, Spain; 3 Ministry of Health and Social Services, P/Bag 13198, Windhoek, Namibia

**Keywords:** Workload Indicators of Staffing Need, WISN, Human resources, Health workforce, Workload, Namibia

## Abstract

**Introduction:**

As part of ongoing efforts to restructure the health sector and improve health care quality, the Ministry of Health and Social Services (MoHSS) in Namibia sought to update staffing norms for health facilities. To establish an evidence base for the new norms, the MoHSS supported the first-ever national application of the Workload Indicators of Staffing Need (WISN) method, a human resource management tool developed by the World Health Organization.

**Application:**

The WISN method calculates the number of health workers per cadre, based on health facility workload. It provides two indicators to assess staffing: (1) the gap/excess between current and required number of staff, and (2) the WISN ratio, a measure of workload pressure. Namibian WISN calculations focused on four cadres (doctors, nurses, pharmacists, pharmacy assistants) and all four levels of public facilities (clinics, health centers, district hospitals, intermediate hospitals). WISN steps included establishing a task force; conducting a regional pilot; holding a national validation workshop; field verifying data; collecting, uploading, processing, and analyzing data; and providing feedback to policy-makers.

**Challenges:**

The task force faced two challenges requiring time and effort to solve: WISN software-related challenges and unavailability of some data at the national level.

**Findings:**

WISN findings highlighted health worker shortages and inequities in their distribution. Overall, staff shortages are most profound for doctors and pharmacists. Although the country has an appropriate number of nurses, the nurse workforce is skewed towards hospitals, which are adequately or slightly overstaffed relative to nurses’ workloads. Health centers and, in particular, clinics both have gaps between current and required number of nurses. Inequities in nursing staff also exist between and within regions. Finally, the requirement for nurses varies greatly between less and more busy clinics (range = 1 to 7) and health centers (range = 2 to 57).

**Policy implications:**

The utility of the WISN health workforce findings has prompted the MoHSS to seek approval for use of WISN in human resources for health policy decisions and practices. The MoHSS will focus on revising staffing norms; improving staffing equity across regions and facility types; ensuring an appropriate skill mix at each level; and estimating workforce requirements for new cadres.

## Introduction

Namibia is an upper-middle-income country characterized by one of the greatest income inequalities in the world, with a Gini coefficient of 0.6 in 2009 to 2010 (where 1 represents complete inequality) [1,2]. Although equity has been a guiding principle of the country’s primary health care strategy since 1990 [3], and access to health services in both rural and urban settings has improved, historically skewed resource allocation [4] affects the distribution of health workers and quality of health service delivery.

In recent years, public dissatisfaction with health care quality has increased considerably, alongside concerns over high maternal and child mortality. A report by the Presidential Commission of Inquiry into Namibia’s Health Service, presented to Parliament in March 2013 [5], highlighted the severe shortage of health professionals as one of the main reasons for poor health outcomes. In addition to health worker shortages, the country’s health care system also faces problems of inadequate local training capacity, uncompetitive salaries and benefits, relatively high attrition rates, and low staff motivation [5].

The Presidential Commission recommended prompt implementation of efforts initially launched in 2009 to restructure the Ministry of Health and Social Services (MoHSS). At that time, the Ministry formed a restructuring task force (RTF). As part of these ongoing restructuring efforts, the country’s Public Service Commission (PSC) charged the Ministry with updating the staffing norms for all operational health facilities to enable informed assessment of any proposed new staff positions. Seeking a means of establishing an evidence base for the new staffing norms, the MoHSS and RTF supported the application of the Workload Indicators of Staffing Need (WISN) method in Namibia.

The WISN method is a versatile human resource management tool developed and later revised by the World Health Organization (WHO) [6,7]. It has been used in a number of different settings and a variety of countries [8-14]. In the first-ever application of WISN at the national level, Namibia used the method to calculate the required number of health workers (for four cadres) in all public sector health facilities. In this paper, we describe the national application of the WISN method in Namibia and explore some of the challenges encountered. We also describe key findings and highlight their use in human resources for health (HRH) policy-making in Namibia.

### Application of WISN in Namibia

The WISN method calculates the number of health workers per cadre, based on the workload of a particular health facility. It provides two basic indicators to assess a staffing situation: (1) the gap/excess between current and required number of staff and (2) the WISN ratio, a measure of workload pressure on health workers. Managers can use WISN findings to compare staffing of similar facilities in a single administrative area (for example, health centers in one district) or contrast staffing levels of a particular cadre between different types of health facilities and different administrative areas. (For further details on the WISN method and process, see text box).

The original impetus for using the WISN method in Namibia came from the chief medical officer for the Kavango region, who approached the MoHSS for permission to pilot the WISN method to estimate the region’s staff requirements. The MoHSS gave its consent and later made the decision to extend WISN to the national level since the findings from Kavango were perceived to be beneficial to the restructuring process. With support from IntraHealth International and the US Agency for International Development (USAID), both the Kavango and national WISN calculations focused on four categories of health workers: doctors, nurses, pharmacists, and pharmacy assistants. The regional and national analyses encompassed all levels of public sector facilities, including clinics, health centers, district hospitals, and intermediate (referral) hospitals.

The Namibian WISN application consisted of the six steps described in the following paragraphs: establishing a WISN task force; piloting the method in Kavango region; conducting a national validation workshop; performing field verification of the data; collecting, uploading, processing, and analyzing the data; and providing feedback to senior MoHSS policy-makers and managers.

### WISN technical task force

Staff members of the MoHSS and IntraHealth set up a 12-member WISN technical task force, which implemented both the Kavango regional pilot and the national application of WISN. This task force comprised senior MoHSS leaders (Policy, Planning, Human Resources Development and Human Resources Management) and key regional figures including the chief medical officer, senior nurses, and human resources experts. The task force also was responsible for briefing members of the MoHSS RTF and other policy-makers and managers.

### Kavango regional pilot

Groups of knowledgeable doctors, nurses, and pharmacy staff came together in two-day workshops to determine the main workload components of each cadre and define activity standards. For this WISN exercise, the steering committee requested that the groups list the activities that a cadre *should* be performing given adequate staffing (rather than those it was currently performing) in order to estimate the number in each cadre required to do this work. This distinction is important because severe shortages of some cadres have forced other cadres, especially nurses, to shoulder additional work and take on responsibilities outside of their expected job descriptions. It would not be possible to improve this situation if we included the additional activities of overburdened cadres in the WISN calculations.

### National validation workshop

Over 100 participants attended a two-day national workshop to validate the main workload components and activity standards established by the Kavango regional pilot. Workshop participants included the four cadres (that is, senior doctors, nurses, pharmacists, and pharmacy assistants) and represented 12 of the 13 regions, key departments, and divisions of the MoHSS as well as other relevant partners. Participants first refined the activity lists and standards in their respective professional groups after identifying several missing activities and next worked to reach consensus in multiprofessional groups arranged by management level and subsequent plenary. Bringing together diverse cadres engendered productive discussions regarding staff activities and overlap.

### Field verification of data

Using routinely collected data to support analyses that inform policy and management decisions strengthens data quality at the national level. Accordingly, the WISN process collected only minimal primary data and used nationally available sources of health and human resources information. Each public sector health facility reports data on a monthly basis into several district and regional databases that are subsequently submitted into national-level databases: the Health Information System database (HIS), the electronic patient management system (ePMS) for HIV/AIDS-related data, and the pharmaceutical management information systems (PMIS and EDT). To ensure credibility of the WISN findings, the WISN task force verified a sample of the nationally available data by comparing them with sources of primary data. Task force members visited four geographically distinct regions (Erongo, Karas, Omaheke, and Omusati) to collect primary service statistics and staffing data from selected health facilities. Task force members found no sizable discrepancies between the primary-level and national-level data from the databases. This assured us that the national databases could provide reliable workload estimates and did not over- or underreport compared with the primary data we collected.

### Data collection, upload, processing, and analysis

Prior to carrying out any data entry, the WISN task force examined the final lists of main activities and associated activity standards elaborated at the national validation workshop. Task force members reviewed both lists for consistency between cadres and facility types and deleted several health service activities where national service statistics were unavailable. These activities were then called either support or additional activities, depending on whether the activity was undertaken by all or only some providers of a particular cadre; a category or individual allowance was then set for each activity. For example, all doctors routinely do small procedures such as electrocardiograms or scans, but because they are not recorded in the HIS database this activity was switched from a health service activity to a support activity. Table 1 provides an example of the main health service activities and activity standards for a nurse at a health center or clinic and defines each workload data item.

**Table 1:** Service standards for nurses in health centers and clinics, Namibia 2012

**Activities**	**Service standards for health centers**	**Service standards for clinics**	**Workload data for the most recent fiscal year**
Admit a patient	20 minutes/admission	n/a	Total admissions
Discharge a patient	5 minutes/discharge	n/a	Total discharges
Death: last office	60 minutes/death	n/a	Total deaths
Conduct a daily ward round	10 minutes/inpatient	n/a	Total admissions^a^
Routine nursing care	30 minutes/inpatient	n/a	Total admissions
Give injections	10 minutes/injection	10 minutes/injection	Total injections, other than immunizations and family planning (FP)
Take lab specimens, including for antiretroviral therapy (ART)	10 minutes/lab specimen	10 minutes/lab specimen	Total lab specimens taken, including ART
Perform a dried blood spot (DBS) test	10 minutes/DBS test	10 minutes/DBS test	Total DBS tests done
Monitor and manage normal delivery	240 minutes/normal delivery	n/a	Total normal deliveries^b^
Monitor and manage emergency delivery	180 minutes/emergency delivery	180 minutes/emergency delivery	Total emergency deliveries
Immediate post-natal care of mother and baby	60 minutes/delivery	60 minutes/delivery	Normal + forceps + vacuum + emergency deliveries + born before arrival
Screen and treat outpatients	25 minutes/outpatient	25 minutes/outpatient	Total outpatients (OPD first visits and revisits)
Outpatient department (OPD) procedure	25 minutes/procedure	25 minutes/procedure	Total OPD procedures
Take a pap smear	20 minutes/pap smear	20 minutes/pap smear	Total pap smears
Antenatal care (ANC) first visit	30 minutes/ANC first visit	30 minutes/ANC first visit	Total ANC first visits
ANC revisit	20 minutes/ANC revisit	20 minutes/ANC revisit	Total ANC revisits
Post-natal visits	30 minutes/post-natal visit	30 minutes/post-natal visit	Total post-natal visits
Growth monitoring of children	10 minutes/child	10 minutes/child	Total children growth monitored
Immunization of women of reproductive age	10 minutes/immunization	10 minutes/i006Dmunization	Total tetanus toxoid immunizations for women aged 15 to 49 years - all doses
Immunization of children under one year old	10 minutes/immunization	10 minutes/immunization	Total immunizations of under-one year-olds - all vaccines, all doses
Family planning first visit	15 minutes/FP first visit	15 minutes/FP first visit	Total FP first visits
Family planning revisit	10 minutes/FP revisit	10 minutes/FP revisit	Total FP revisits
Integrated management of adult illness (IMAI)	30 minutes/HIV/AIDS revisit	30 minutes/HIV/AIDS revisit	80% of the total HIV/AIDS revisits plus 80% of enrolled in HIV/AIDS care and treatment^c^
Pre-test counseling of voluntary counseling and testing (VCT) clients	15 minutes/VCT client	15 minutes/VCT client	10% of the total VCT clients pre-test counseled
Post-test counselling of VCT clients	30 minutes/VCT client	30 minutes/VCT client	10% of the total VCT clients post-test counseled
Provide prevention of mother-to-child transmission (PMTCT) counseling and testing	10 minutes/client	10 minutes/client	Total clients who received PMTCT
Provide TB DOTS	10 minutes/DOTS visit	10 minutes/DOTS visit	40% of the total DOTS visits
Provide ART care and treatment	15 minutes/ART revisit	15 minutes/ART revisit	20% of total ART revisits plus 20% of enrolled in care and treatment
Dressing wounds	10 minutes/dressing	10 minutes/dressing	Total dressings

^a^Health centers do not keep data on patient days since a client stay cannot exceed 48 hours. Therefore using the number of admissions as the data source gives an estimate for daily ward rounds and routine nursing care at a health center.

^b^Clinics only provide emergency deliveries; normal deliveries should occur at hospitals and certain health centers.

^c^Several activities are done by more than one cadre; the percent represents the proportion of time the specific cadre conducts that activity.

*ANC*: antenatal care; *ART*: antiretroviral therapy; *DBS*: dried blood spot; *DOTS*: directly observed treatment, short-course; *FP*: family planning; *IMAI*: integrated management of adult illness; *OPD*: outpatient department; *PMTCT*: prevention of mother-to-child transmission; *VCT*: voluntary counseling and testing.

A small group of MoHSS staff ensured availability of relevant data from national data sources and uploaded or entered the data to the WISN software during a two-day retreat. Although the WISN software does not currently support automatic upload of data, this was made possible through development of a small software program to upload the data.

### Feedback to senior MoHSS policy-makers and managers

The WISN task force regularly briefed the MoHSS RTF on the progress of both the WISN pilot and the national application. At the request of the RTF, the WISN task force also made several high-level presentations to senior managers, including presentations of WISN findings at the MoHSS National Management Development Forum (February 2013) and the MoHSS Strategic Management Retreat (July 2013). WISN task force members provided regular progress briefings to the Ministerial Steering Committee chaired by the Minister of Health.

### Challenges in applying the WISN method

The WISN task force faced two main types of challenges in applying the WISN method. The first set of challenges resulted from the WISN software itself, while the second concerned the lack of availability of particular data items at the national level.

### Software challenges

The WHO-developed WISN software is not open source. As a result, we were not able to examine in detail how the software performs its calculations. This created several challenges. For example, two separate reports initially provided different answers for the same calculated requirement. This was brought to WHO’s attention and later corrected in an updated version of the software. Another software-related challenge was related to the lack of clear direction in the WISN software manual regarding how the software handles workload activities that are needed 24 hours a day and 365 days a year. Through manual calculations, we determined that the software does not adjust automatically for such activities.

### Data challenges

Data on a few important workload components (such as the number of major and minor operations) were available at the facility level but not at the national level. Because the task force considered those workload activities critical to account for the full workload of relevant cadres, the team collected the missing data directly from hospital theater registers. Additionally, the number of patient days is an important indicator of workload; because the HIS database only inputs the number of patient discharges, patient days had to be calculated from available midnight census report data.

### WISN findings

This paper presents key findings of particular relevance to the use of the WISN method to inform HRH policy and practice in Namibia. Although we discuss results for other cadres as well, we particularly emphasize findings as they relate to nurse staffing because nurses form the country’s biggest group of health workers (282 doctors versus 4,251 nurses working in the public sector).

The core WISN findings in Namibia can be summed up in two words: shortage and inequity. Overall, staff shortages are most profound for doctors and pharmacists. Both the intermediate and district hospitals have only one-third of the doctors that they require based on workload. The shortage of pharmacists is even more severe. These serious staffing gaps are not a surprise because Namibia is still reliant on overseas recruitment for both cadres. The local training programs have yet to graduate the first Namibian students.

The WISN analysis found that only seven pharmacy assistants work in health centers, representing 11% of the workload-based requirement. The analysis found no pharmacy assistants at the clinic level. Even at the district hospital level, Namibia has only about a third of the pharmacy assistants that the facilities’ current workload would require.

The WISN analysis of nurse staffing shows that on the whole the country has an appropriate number of nurses. The nurses are, however, very inequitably distributed between the different types of facilities. The total nurse workforce in Namibia is clearly skewed towards hospitals. This type of inequity exists, of course, in many countries in Africa and elsewhere and represents health professionals’ desire to live in urban environments offering better amenities for themselves and their families [15]. The WISN results for nurses showed that both intermediate and district hospitals are adequately or even slightly overstaffed relative to their workloads (Table 2), with excesses of 121 and 148 nurses at intermediate and district hospitals, respectively. However, while 18 of the 29 district hospitals have more nurses than they require on the basis of their workload, 10 actually have a shortage. At the health center level, health centers have only 85% of their required nursing staff, representing a gap of 63 nurses. In clinics, nursing staffing is only 77% of what is required, representing a gap (210 nurses) that is over three times larger than the gap for health centers. It should be noted that the health center shortage would appear greater if the facilities operated around the clock as intended. However, many - probably most - health centers operate only during the daytime due to insufficient staff.

**Table 2:** Overall nurse staffing by type of health facility, Namibia 2012

**Type of health facility**	**Number of health facilities**	**Current number of nurses**	**Required number of nurses**	**Gap/excess**	**% of requirement**
Intermediate hospitals	5	1,621	1,500	121	108%
District hospitals	29	1,542	1,393	148	111%
Health centers	38	363	426	-63	85%
Clinics	278	693	903	-210	77%
Total	350	4,219	4,223	-4	100%

Considerable inequity exists between and within regions. At the health center level, for example, Ohangwena region had no nurses in its only health center though calculations showed that it required 21 (Table 3). According to the WISN ratio (a proxy measure for workload stress), health center nurses in Omusati region are under the greatest workload stress (WISN ratio of 0.13), while those in Karas region experience the least (WISN ratio of 2.46). Expressing these WISN ratios another way, health centers in Karas have 246% of their nurse requirement, while Omusati health centers have only 13%. The WISN method also makes it possible to analyze staffing equity between health centers in one region (provided the region has more than one health center) or between clinics. In Oshana region, for example, two of the five health centers have excess nursing staff. The best staffed center has 160% of its required staffing, while the three understaffed ones have only about 40% of their requirement. Regional variation also can be illustrated by ranking regions by the size of the gap or excess in their required staffing for specific facility types and cadres. For example, Table 4 shows that the first four regions account for 84% of the total nurse staffing gap (177/210 nurses) at the clinic level.

**Table 3:** Equity between regions in health center (HC) nurse staffing, ranked by WISN ratio, Namibia 2012

**Region**	**Number of HCs**	**Current # of nurses**	**Required # of nurses**	**Gap/excess of nurses**	**WISN ratio for nurses**
Ohangwena	1	0	21	-21	0.00
Omusati	7	8	61	-53	0.13
Otjozondjupa	3	16	25	-9	0.65
Caprivi	3	11	16	-5	0.70
Khomas	2	51	73	-22	0.70
Oshana	5	73	90	-17	0.81
Omaheke	1	6	7	-1	0.88
Erongo	1	18	16	2	1.14
Kavango	7	78	63	15	1.25
Oshikoto	3	34	25	9	1.37
Hardap	3	47	22	25	2.11
Karas	2	21	9	12	2.46
Total	38	363	426	-63	0.85

**Table 4:** Equity between regions: clinic staffing by nurses ranked by the gap/excess, Namibia 2012

**Region**	**Number of clinics**	**Current # of nurses**	**Required # of nurses**	**Gap/excess**	**WISN ratio**
Ohangwena	30	105	158	-53	0.66
Kavango	48	83	130	-47	0.64
Kunene	24	13	55	-42	0.24
Omusati	39	84	119	-35	0.71
Oshikoto	20	52	72	-20	0.72
Omaheke	12	24	39	-15	0.61
Erongo	17	57	72	-15	0.79
Otjozondjupa	16	42	56	-14	0.75
Oshana	11	23	35	-12	0.67
Hardap	12	30	35	-5	0.87
Karas	14	47	49	-2	0.97
Caprivi	26	73	55	18	1.33
Khomas	9	60	30	30	2.02
Total	278	693	903	-210	0.77

The analysis of WISN findings demonstrates that workloads can vary widely within the same health facility type. Moreover, several clinics cope with workloads that are higher than some health centers. In response to these variable workloads, the workload-based requirement for nurses in the 278 clinics ranges from less than one nurse per clinic to over 17. In the 38 health centers, the range is from a little over two nurses required in less busy centers to over 57 in the busiest.

### Using the WISN findings to inform HRH policy

Namibia’s vast distances and relatively low population densities create considerable challenges for MoHSS efforts to balance health care equity, efficiency, and quality. Furthermore, the political pressure for enhanced quality has risen in the aftermath of the recommendations of the Presidential Commission of Inquiry. The evidence-based WISN results provide important information for the MoHSS as it seeks to improve quality without losing sight of other considerations.

The utility of the health workforce findings generated by the national application of the WISN method has prompted the MoHSS to seek cabinet approval for the wider use of WISN in HRH policy decisions and practices. In the short term, the Ministry will focus its efforts on three key areas: revising staffing norms; improving staffing equity across regions and types of facilities; and ensuring an appropriate skill mix at each level, including estimating workforce requirements for new cadres. At a later stage, the MoHSS intends to use the WISN method to model future staff requirements based on different assumptions about workloads and other key factors such as training outputs, changing demographics and disease profiles, and staff turnover.

Although no official guidelines exist about how often to rerun the WISN calculations, we recommend running new WISN estimates every two to three years, depending on the budget cycle. The workload components should be reassessed every five or six years to ensure that they continue to reflect current activities and the standards for completing those activities. If health systems add new cadres or implement task-sharing, the WISN method should be used to estimate the new workload for those specific cadres.

### Revising staffing norms

Namibia’s PSC requires a detailed facility-level proposal on staffing norms to approve staff positions in public sector facilities. However, the current staffing norms in Namibia, as in most countries in the region, are not related to the workload requirements of a specific facility. Rather, they are based on a set number of staff by cadre according to the type of health facility. The WISN findings can be used in different ways to define more appropriate staffing norms. Although one approach might be to use the average staff requirement as the new staffing norm, the WISN findings demonstrate that health centers and clinics in Namibia vary widely in their workloads. This implies that two types of clinics would require two different staffing norms: a small clinic might require only one or two nurses whereas a large clinic with a heavy workload could require up to 17. Another approach to setting staffing norms is to base the number of approved posts for a given facility on the WISN calculations of the most recent year’s workload data for that facility. However, this approach ignores staffing requirements for services that the facility should be, but currently is not, delivering due to staff shortages. Therefore, the MoHSS will recommend to the PSC an approach for setting the new staffing norms that integrates two important considerations not previously addressed: the skill mix of cadres required to provide the minimum service package for a given facility type, and the workload pressure on the staff.

### Equity in staffing in relation to workloads

It is possible to sort WISN findings by type of facility and cadre and easily identify the facilities most in need of additional staff. By pinpointing the health facilities with the highest workload pressure and improving their staffing, the MoHSS expects to see improved quality of care. It also plans to make immediate improvements in nurse staffing in health facilities falling below an identified cut-off point (a WISN ratio of 0.6 or less, meaning 60% or less of required staffing).

Staffing can be improved both by creating new posts and transferring existing staff. The process of staff transfers remains centralized through the PSC. A transfer requires a vacant staff post in the receiving facility, necessary budgetary provision for the post, and the consent of the individual to be transferred. WISN findings can be used to advocate for transfers (and, where necessary, new posts) that would greatly improve staffing equity in a region. Using the example of Karas region, Table 5 shows how the WISN findings could be used to identify transfer options to alleviate workload pressure on nurses in some clinics and address overstaffing in others. The transfers would address nurse understaffing in the Berseba, Daan Viljoen, Oranjemund, and Rosh Pinah clinics (which currently have only about a third to a half of the number of nurses they require) and lessen overstaffing in clinics such as Warmbad and Noordoewer, which have two to four times their nurse staffing requirement.

**Table 5:** Equity within one region before and after transfers: clinic nurse staffing, Karas region, Namibia 2012

**Clinic**	**Current # of nurses**	**Required # of nurses based on WISN**	**# of nurses after transfer**	**WISN ratio before transfer**	**WISN ratio after transfer**
Ariamsvlei	2	1.2	2	1.69	1.69
Aus	2	1.5	2	1.33	1.33
Aussenkher	2	2.7	2	0.75	0.75
Berseba	1	2.2	2	0.45	0.90
Daan Viljoen	2	5.7	5	0.35	0.88
Karasburg	5	4.0	4	1.24	1.00
Keetmanshoop	8	7.4	8	1.08	1.08
Koes	2	2.0	2	0.99	0.99
Lüderitz	7	7.8	7	0.90	0.90
Noordoewer	7	3.0	3	2.36	1.01
Oranjemund	2	3.9	3	0.52	0.77
Rosh Pinah	2	4.4	4	0.46	0.91
Tses	2	2.2	2	0.90	0.90
Warmbad	3	0.7	1	4.17	1.39
Total	47	48.6	47	0.97	0.97

A staff requirement of less than one raises a question of whether the facility should be closed because of its low workload. However, WISN findings alone are not sufficient for making the decision to shut down a facility. Decisions of this nature require thorough knowledge of local health needs and access to services. Given Namibia’s sparse population and access difficulties, there invariably will be a trade-off between equity and efficiency.

### Skill mix

Although this paper focuses predominantly on the WISN findings for nurses, their staffing situation must be considered relative to other relevant cadres. The WISN results demonstrate a severe shortage of doctors and pharmacists in every district hospital, with 196 fewer doctors than required but an excess of 148 nurses (Table 6). Because these district hospital posts for doctors are unlikely to be filled any time soon, it is important to consider policies that can immediately be put in place to improve staffing and quality of care at these facilities.

**Table 6:** National WISN results for doctors, nurses, pharmacists, and pharmacy assistants, Namibia 2012

**Health facility**	**Doctors**		**Nurses**		**Pharmacists**		**Pharmacy assistants**	
	**Gap**	**WISN ratio**	**Gap**	**WISN ratio**	**Gap**	**WISN ratio**	**Gap**	**WISN ratio**
IH	-70	0.31	-0.1	0.96	-23	0.12	+1	1.16
DH	-196	0.36	+148	1.1	-189	0.03	-99	0.32
HC			-63.2	0.85			-54	0.11
Clinic			-210	0.77			-136	0.02
Total	-266		-125		-212		-288	

*DH*: district hospital; *HC*: health center; *IH*: intermediate hospital.

As their main workload components indicate, doctors and nurses share some activities, such as enrolling patients in antiretroviral therapy (ART) care and treatment, taking pap smears, screening and treating outpatients, and admitting and discharging patients. Each of these workload components can be examined to determine which, from a competency perspective, only a doctor should perform, which could be competently performed by either a doctor or a nurse, and which a nurse could perform with limited additional training. This type of analysis would support an informed decision either to transfer certain tasks to nurses or share tasks between doctors and nurses. (In the latter case, the conditions under which a doctor would still need to see a patient should be defined, even while recognizing that nurses could competently care for most patients.) Nurses in Namibia already undertake some ART care and treatment activities that doctors would carry out in better-staffed settings. For example, ART enrollment is being shifted to nurses with the approval of the Health Professions Council of Namibia, provided that the nurses have successfully completed the Council’s approved training. The responsibility for ART enrollment is expected to transfer over time from doctors to nurses for a majority of cases. After this shift has taken place, it will be important to rerun the WISN calculations to estimate the required staff numbers of doctors and nurses under the new arrangement.

The WISN findings showed that Namibian clinics have variable workloads. Many clinics have a small pharmacy workload and require only between 0.25 and 1.0 pharmacy assistants. Placing a pharmacy assistant in these clinics is not a good use of a scarce human resource, particularly given that dispensing by nurses in these settings is already accepted practice. The MoHSS has accepted a policy to introduce pharmacy assistants to those clinics where the workload warrants such a post, which can be identified on the basis of the current WISN results. Running the WISN calculations again after the dispensing workload has been adjusted can provide updated estimates of pharmacy assistant requirements as well as nurse staffing requirements at clinics.

The MoHSS expects to use the WISN findings in considering the possible introduction of a new staff cadre. Currently, Namibia has no staff category between a nurse and a doctor, although such cadres play key primary care roles in many other countries [16,17]. If a new cadre such as a clinical officer is to be introduced to relieve doctors’ work burden in district hospitals and busy health centers, it would be important to examine doctors’ workload components to determine which activities are most appropriate to transfer to the new cadre. The focus should be on determining the activities that this new cadre can competently perform after appropriate training. After redefining doctors’ workload components and defining workload components for the new cadre, the number of required staff in both categories can easily be estimated by running a new set of WISN calculations.

## Conclusion

The WISN method is a dynamic and useful tool that offers credible workload-based support to national, regional, and local policy-makers and facility managers to improve the equity and distribution of health workers within a region or across similar types of facilities nationwide. Making novel use of existing databases, WISN also can contribute to improved data quality while reducing the need for additional primary data collection to estimate workload requirements. More importantly, the WISN tool allows policy-makers to consider the impact of decisions on staff requirements before actually making them. As the Namibia example suggests, policy-makers and facility managers can use the WISN method to estimate health worker requirements for a range of needs and scenarios, including making staff adjustments in response to implementation of new services, decentralization, or reconfiguration of primary care services.

### Text box: the WISN method and process

The World Health Organization developed the Workload Indicators of Staffing Need (WISN) method in the late 1990s. The goal was to bring human resource planning principles, long used in business and industry, to the health sector. The original WISN user’s manual was published in 1998.^a^ A decade later, much experience had been gained in applying the WISN method. With WHO support, the WISN user’s manual was revised, a set of case studies written, and WISN software and software manual developed. (These materials can be accessed through http://www.who.int/hrh/resources/wisn_user_manual/en/). The revised WISN user’s manual^b^ describes both the WISN method and the WISN process. The *WISN method* is a human resource management tool that calculates a staff requirement based on workload for a particular staff category and type of health facility. This tool can be applied nationally, regionally, or only for a single health facility or even a unit/ward at a hospital, provided relevant service statistics are available.

The steps of the WISN method are:

• Determining the priority cadre(s) and health facility type(s) for applying the WISN method.

• Estimating available working time, defined as the time a health worker has available in one year to do their work, given authorized and unauthorized absences for leave, sickness, and so on.

• Defining workload components, consisting of both health service activities and those supporting these activities (such as recording, reporting, and management meetings).

• Setting activity standards, defined as the time necessary to perform an activity to acceptable professional standards in the local circumstances.

• Establishing standard workloads (that is, the amount of work within a health service component that one health worker can do in a year).

• Calculating allowance factors in order to take account of the staff requirement of support activities performed by all or some of the staff for which there are no service statistics.

• Determining staff requirements based on WISN by calculating the total staff required to cover both health service activities and activities supporting the services.

• Analyzing and interpreting the WISN results.

An analysis of WISN results provides two different measures: (1) the difference between current and required number of staff, and (2) the WISN ratio (current staff divided by required staff). The WISN ratio is a proxy measure for the daily workload pressure on the staff. Examining both the gap or excess in staffing and the WISN ratio is important in determining how to improve staffing equity; a staffing gap of the same size has a much bigger impact on workload stress in a health facility with only a few staff than in one with a large staff.

The *WISN process*, in turn, is the set of activities that are needed to apply the WISN method in practice. They range from mobilizing commitment to eventually integrating the WISN method into routine management systems. The WISN process is flexible and should be designed to fit the goals and scope of the WISN application. Three different sets of individuals are generally required for successful application. The size and composition of these groups will vary between different WISN applications. The first group is a steering committee of senior-level people with authority to influence decision-making based on the WISN results. The second is a technical task force, responsible for the actual application of the WISN method. This task force works with one or several expert working groups. The task of this third set of individuals, the expert working group, is to define the workload components and to set activity standards for their respective cadre(s).

## Endnotes

^a^Shipp P: *Workload Indicators of Staffing Need (WISN). A manual for implementation*. Geneva: World Health Organization; 1998 (WHO/HRB/98.2)

^b^World Health Organization: *Workload Indicators of Staffing Need. User’s Manual*. Geneva: World Health Organization; 2010

## Abbreviations

ANC: Antenatal care; ART: Antiretroviral therapy; DBS: Dried blood spot; DH: District hospital; DOT: Directly observed treatment, short-course; EDT: Electronic dispensing tool; ePMS: Electronic patient management system; FP: Family planning; HC: Health center; HIS: Health information system; HRH: Human resources for health; HRIS: Human resource information system; IH: Intermediate hospital; IMAI: Integrated management of adult illness; MoHSS: Ministry of Health and Social Services; PMCTC: Prevention of mother-to-child transmission; PMIS: Pharmaceutical management information system; PSC: Public service commission; RTF: Restructuring task force; USAID: United States Agency for International Development; VCT: Voluntary counseling and testing; WHO: World Health Organization; WISN: Workload indicators of staffing need.

## Competing interests

The authors declared that they have no competing interest.

## Authors’ contributions

PM participated in all phases of the WISN work, presented the results to key policy-makers at the Ministry of Health and Social Services, contributed to the manuscript’s conceptualization, and drafted the paper’s background and policy sections. RLKA trained the key stakeholders to use the WISN tool nationally, contributed to the manuscript’s conceptualization, drafted the methods and findings sections, and contributed to editing the overall draft. As a key policy-maker in Namibia, NF led the effort to implement the WISN pilot and national application, contributed to the paper’s conceptualization, and drafted background and policy sections to elucidate the Namibian context. All authors read and approved the final manuscript.

## Authors’ information

PM is a registered nurse with a doctorate in social policy. She has worked across Africa over the past fifteen years offering technical assistance and capacity-building support to improve human resources for health strategies. She is the current Chief of Party for IntraHealth International in Namibia and was instrumental in implementing the WISN activities in Namibia.

RLKA is a medical doctor with a doctorate in public health. She works as a consultant in the areas of health and human resource policy formulation, planning, and evaluation, and the governance of health systems in resource-poor countries. She revised the WISN User’s Manual and edited the WISN case studies for WHO. In Namibia, she supported the WISN application as the external consultant.

NF is a medical doctor with a master’s degree in health economics. He is the Deputy Permanent Secretary for the Ministry of Health and Social Services in Namibia and chairs the MoHSS Restructuring Task Force. He was a key policy-maker overseeing the implementation of the WISN exercise.

## References

[B1] The World Bank Data: Namibia[http://data.worldbank.org/country/namibia] (accessed 22 July 2013)

[B2] The World Bank Namibia overview[http://www.worldbank.org/en/country/namibia/overview] (accessed 22 July 2013).

[B3] Ministry of Health and Social Services Towards Achieving Health and Social Well Being for all Namibians: a Policy Framework 1998 Windhoek

[B4] Ministry of Health and Social Services Equity in Health Care in Namibia: Towards Needs-based Allocation Formula 2005 Harare: Regional Network for Equity in Health in Southern Africa (EQUINET) Discussion Paper Volume 26

[B5] Ministry of Health and Social Services Report of the Presidential Commission of Inquiry: Ministry of Health and Social Services to His Excellency, President Hifikepunye Pohamba 2013 Windhoek

[B6] Shipp P Workload Indicators of Staffing Need: a Manual for Implementation 1998 Geneva: World Health Organization

[B7] World Health Organization Workload Indicators of Staffing Need: User’s Manual 2010 Geneva: WHO Press

[B8] Hagopian A Mohanty MK Das A House PJ Applying WHO’s ‘workforce indicators of staffing need’ (WISN) method to calculate the health worker requirements for India’s maternal and child health service guarantees in Orissa State Health Policy Plan 2012 11 11 18 10.1093/heapol/czr007 21296847

[B9] Kolehmainen-Aitken R-L Shipp P ‘Indicators of staffing need’: assessing health staffing and equity in Papua New Guinea Health Policy Plan 1990 11 167 176 10.1093/heapol/5.2.167

[B10] World Health Organization Applying the WISN Method in Practice: Case Studies from Indonesia, Mozambique and Uganda 2010 Geneva: WHO Press

[B11] WISN Tool Workload Indicators of Staffing Need. Applying the WISN Method in Practice Case Studies from Jersey, Newcastle and Derbyshire: WHO/EURO forthcoming

[B12] Musau P Nyongesa P Shikhule A Birech E Kirui D Njenga M Mbiti D Bett A Lagat L Kiilu K Workload indicators of staffing need method in determining optimal staffing levels at Moi Teaching and Referral Hospital East Afr Med J 2008 11 232 239 18814533 10.4314/eamj.v85i5.9617

[B13] Ozcan S Hornby P Determining hospital workforce requirements: a case study Int J Health Plann Manage 1995 11 305 319 10.1002/hpm.4740100406 10154308

[B14] Hossain B Alam SA Likely benefit of using Workload Indicators of Staffing Need (WISN) for human resources management and planning in the health sector of Bangladesh Hum Resour Health 1999 11 99 111

[B15] World Health Organization Increasing Access to Health Workers in Remote and Rural Areas Through Improved Retention: Global Policy Recommendations 2010 Geneva: WHO Press 23741785

[B16] Dovlo D Using mid-level cadres as substitutes for internationally mobile health professionals in Africa. A desk review Hum Resour Health 2004 11 7 18 10.1186/1478-4491-2-7 15207010 PMC455693

[B17] Alliance GHW Mid-level Health Providers: a promising resource to achieve the health millennium development goals 2010 Geneva: WHO Press

